# Selection of Native Tree Species for Subtropical Forest Restoration in Southwest China

**DOI:** 10.1371/journal.pone.0170418

**Published:** 2017-01-19

**Authors:** Yang Lu, Sailesh Ranjitkar, Rhett D. Harrison, Jianchu Xu, Xiaokun Ou, Xuelan Ma, Jun He

**Affiliations:** 1 Key Laboratory for Plant Diversity and Biogeography of East Asia, Kunming Institute of Botany, Chinese Academy of Sciences, Kunming, Yunnan, China; 2 University of Chinese Academy of Sciences, Beijing, China; 3 Institute of Ecology and Geobotany, Yunnan University, Kunming, Yunnan, China; 4 World Agroforestry Centre, ICRAF East and Central Asia, Kunming, Yunnan, China; 5 World Agroforestry Centre, East and Southern Africa Region, Lusaka, Zambia; 6 National Centre for Borderland Ethnic Studies in Southwest China, Yunnan University, Kunming, Yunnan, China; 7 School of Ethnology and Sociology, Yunnan University, Kunming, Yunnan, China; Pacific Northwest National Laboratory, UNITED STATES

## Abstract

The use of native species in forest restoration has been increasingly recognized as an effective means of restoring ecosystem functions and biodiversity to degraded areas across the world. However, successful selection of species adapted to local conditions requires specific knowledge which is often lacking, especially in developing countries. In order to scale up forest restoration, experimental data on the responses of native species to propagation and restoration treatments across a range of local conditions are required. In this study, the restoration potential of 34 native tree species was evaluated based on nursery research and field planting experiments at a highly degraded site in a subtropical area of southwest China. We examined species performance in terms of germination rates as well as survival rates and growth over 2 years after planting. Of the 34 species examined, 25 had a germination percentage greater than 50%. Survivorship ranged from 0 to 97% across species and was greater than 50% for 20 species. Mean monthly growth increments varied between species. Pioneer species performed well, and 14 mid- and late-successional species performed reasonably well to very well in this study. However, the remaining 16 mid- and late-successional species performed poorly. These results indicate that carefully selected mid- and late-successional species can be effectively incorporated into mixed species plantings. This data can be used to inform restoration planning, helping to identify suitable species and so enhance the biodiversity and resilience of restored forests.

## Introduction

Biodiversity conservation, ecosystem goods and services, and forest-based livelihoods are increasingly threatened by deforestation and forest degradation around the world [1]. The recent results of the Global Forest Resources Assessment 2015 indicate that natural forest area declined from 3,961 to 3,721 million hectares globally between 1990 and 2015 [2]. Forest restoration is now regarded as a cornerstone of global biodiversity conservation and sustainable development [3, 4]. The Bonn Challenge, an implementation platform for many existing international commitments that require restoration, including the Convention on Biological Diversity Aichi Target 15, the UN REDD+ program and the New York Declaration on Forests, has set a target of restoring 150 million hectares of forest worldwide by 2020 and 350 million hectares by 2030 [5, 6]. While these political commitments have garnered wide support, there are huge knowledge gaps if we are to achieve the ambitious restoration targets so far agreed [6], and a set of efficient restoration approaches will be necessary to achieve these targets [7].

To date, the standard paradigm for reforestation in many areas across the world has been to plant monoculture plantations which rely on a relatively small number of common and commercial species [8]. Although such plantations can rapidly increase forest cover, this approach has been widely criticized. Monoculture plantations may increase risks associated with pest and pathogen outbreaks, provide few benefits for ecosystem services, may increase vulnerability to market volatility, and threaten the conservation of many forest-dependent species [9, 10]. In the face of global environmental changes, there is an urgent need for more research on forest restoration using a diversity of species in order to safeguard the function and resilience of restored forests [11, 12].

The published literature on forest restoration includes studies that demonstrate how planting mixtures of native species can restore ecosystem functions, conserve biological diversity, and diversify forest products in degraded landscapes (e.g. [13–17]). These studies emphasize that the most critical step in such restoration plantings is to select suitable native species from the regional species pool. However, the ecological information necessary to select species which are appropriate and adapted to local conditions is inadequate in many places, particularly in developing countries. This lack of data has limited the success of forest restoration programs [17, 18]. To address this knowledge gap, research on the restoration potential of diverse native tree species is urgently needed.

The selection of native species for ecological restoration is much more complex and challenging than the selection of plant materials for monoculture plantations [19, 20]. Consideration of functional groups enables a more rapid categorization of species, especially in highly diverse forests [21, 22]. Plant functional groups are assemblages of species showing similar responses to the environment and similar effects on ecosystem processes, and can be a useful tool in selecting optimal species for restoration [23, 24]. In particular, species successional status can be a means for assigning relevant functional groups to restoration activities [25–27]. Several methods of forest restoration using native species exist at present. Knowles and Parrotta [28] document the maximum diversity method, which uses a large number of later successional species to initiate the succession. This approach is technically challenging and expensive. By contrast, the interplanting of selected pioneer species and later successional species can be used as a simpler and cheaper method of reestablishing the appropriate successional trajectory at degraded sites [25, 29–31]. The pioneer species can act as nurse species [32], which quickly establish a protective tree canopy, shade out competing weeds, and facilitate the establishment of species from later successional stages. One such approach is the framework species method, which involves the planting of early and later successional tree species at the same time, and which has been shown to be an efficient method of forest restoration in Australia and Thailand [13, 33]. However, this method has so far only been shown to be effective for small scale plantings, because it requires consideration of a series of ecological properties of tree species (such as weed suppression, attraction of seed dispersal agents, and tolerance of fire), and knowledge about such traits is often limited [13]. If forest restoration is to be scaled up, restoration planting needs to be a tool that is readily accessible to local communities, including those in developing countries with little financial and technical assistance. In the future, providing simple, evidence-based methods for native tree species selection will support and facilitate local restoration initiatives.

Recent research on species selection has primarily focused on the restoration of biologically rich tropical forests [13, 27, 28, 34, 35]. However, subtropical forests also cover large areas in many countries, such as China, Japan, Australia, USA, Mexico, Argentina, and Brazil; they support globally significant levels of biodiversity; and they provide ecosystem services to a substantial proportion of the world’s population [36, 37]. East Asian evergreen broad-leaved forests are the most extensive type of subtropical forest in the world, but are highly deforested and fragmented due to human activities, particularly in China [38, 39]. At present, Chinese natural subtropical evergreen broad-leaved forests exist only in nature reserves or remote, isolated areas. Such forests are dominated by species of *Lithocarpus*, *Castanopsis*, and *Cyclobalanopsis* (Fagaceae), *Cinnamomum* and *Machilus* (Lauraceae), *Schima* (Theaceae), *Magnolia*, *Manglietia*, and *Michelia* (Magnoliaceae) [40, 41].

Many countries in subtropical regions have implemented reforestation and restoration programs with the aim of restoring native forests and improving ecosystem services [42–45]. In the Chinese subtropics, for example, large-scale state reforestation programs (e.g. the Sloping Land Conversion Program and the Natural Forest Protection Program) have been implemented over recent decades, and forest cover has increased substantially [46]. However, as in other areas of the tropics, most reforestation efforts in the region have established monoculture plantations comprised of a relatively small set of fast-growing commercial species, mainly from the genera *Pinus*, *Cunninghamia*, and *Eucalyptus* [46–48]. This may be at least partly due to the fact that, although the use of a wider range of native species is increasingly encouraged in reforestation and restoration programs [49, 50], existing knowledge of tree species selection techniques for subtropical forests is limited. Principles of industrial forestry techniques are emphasized in the training program for the employees of the forest departments and the employees are often unaware how to incorporate native species restoration into their programs [24, 50, 51]. However, some studies have investigated subtropical forest succession [26, 38, 39, 41], making it possible to assign species to successional guilds. Research on the restoration performance of subtropical tree species from different successional guilds and identification of appropriate species for restoration is essential to support large-scale planting.

We conducted a forest restoration study at Gaoligong Mountains National Nature Reserve, Yunnan Province in southwest China. We examined the performance of 34 native subtropical tree species through nursery research and experimental field trials. Quantitative indicators such as seed germination, seedling early survival and growth rates were combined in order to identify appropriate species. Our study provides information relevant to the selection of species for the restoration of subtropical evergreen broad-leaved forests for the 34 species studied, and by extension to all of the dominant tree genera in this forest type. In addition, the methods described are simple and could be replicated by restoration practitioners elsewhere in order to expand the information on local species.

## Methods

### Ethics statement

This research did not involve human or other animal subjects. For plant collections, we collected the minimum number of specimens necessary to ensure that appropriate vouchers were obtained. The field studies did not involve endangered or protected species. Permission to work in Gaoligong Mountains National Nature Reserve was obtained through a cooperative agreement between Kunming Institute of Botany, Chinese Academy of Sciences and the Management Bureau of Gaoligong Mountains National Natural Reserve.

### Study area

**Fig 1:**
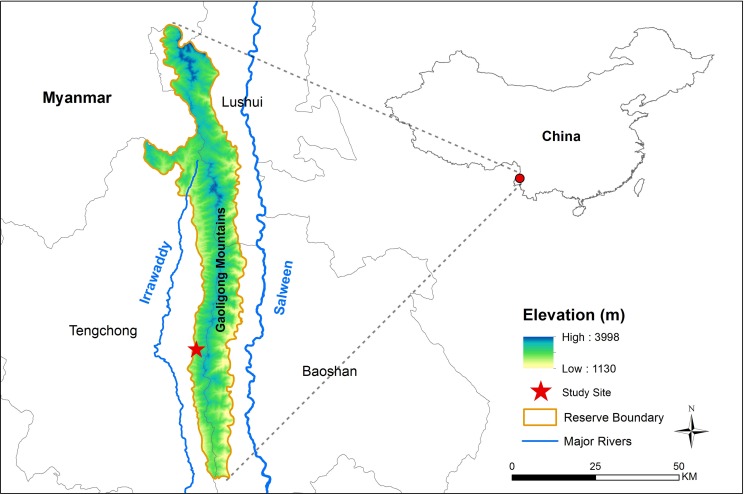
Map of the study site in the Gaoligong Mountains Nature Reserve (southern part) in Yunnan Province, Southwest China.

This research was designed as a small-scale project to develop an appropriate approach for native species selection in forest restoration. Fieldwork was conducted in the southern part of Gaoligong Mountains National Nature Reserve (lat 24°56′ to 28°22′ N, long 98°08′ to 98°50′ E), Yunnan Province in southwest China, located along the border of China and Myanmar (Fig 1). The wet season in this region is influenced by the Indian Ocean Monsoon and occurs from May to October, whereas the dry season runs from November until the following April. The average annual rainfall is approximately 1,977 mm, nearly 80% of which normally falls during the wet season. The monthly mean maximum and minimum temperature are 19.9°C in August and 7.3°C in January, respectively [52]. The Gaoligong Mountains form the drainage divide between the upper Salween and Irrawaddy rivers, providing huge watershed protection services. The Gaoligong Mountains Reserve is also a flagship protected area located within the Eastern Himalayan global biodiversity hotspot [53]. The reserve harbors large areas of well-developed subtropical evergreen broad-leaved forests, which support high phylogenetic diversity and have been identified as a conservation priority [54]. This region is also an environmentally sensitive area and one in which poverty is prevalent. The majority of local residents around the reserve are farmers whose livelihoods are dependent to some degree on the forests. As with many other protected areas in the world [55], the forests of Gaoligong Mountains Reserve have become increasingly isolated as the protected area has been gradually surrounded by plantations and farmland, threatening long-term conservation goals [56]. Forest restoration in and around the reserve can help to provide a buffer and increase the resilience of the reserve. Meanwhile, natural forest in the reserve can provide reference sites and a species pool for restoration practices.

### Species studied

Species were initially selected from native plant communities based on forest ecological knowledge, traditional uses, and availability of seeds. A list of 34 tree species was drawn up from 18 different families and 30 genera (Table 1). Important families included the Fagaceae, Lauraceae, Magnoliaceae, and Rosaceae. Nomenclature follows that of Wu et al. [57]. The local use of native species was surveyed through a traditional ethnobotanical approach [58]. The habitats of the selected species were also documented [52]. The studied species were further classified by their forest successional status based on a literature review [41, 52, 59], expert consultations, and the authors’ own field experience (Table 1).

**Table 1:** Information on 34 native tree species.

Species	Family	Uses^a^	Vegetation^b^	Successional status^c^
*Acer oliverianum* Pax	Aceraceae	OR, TB	P	MS
*Actinodaphne obovata* Blume	Lauraceae	MD, OL, TB	P	LS
*Alcimandra cathcartii* Dandy	Magnoliaceae	OR, TB	P	LS
*Alnus nepalensis* D. Don	Betulaceae	FW, TB	P, S, A	ES
*Betula alnoides* Buchanan-Hamilton ex D. Don	Betulaceae	TB	S	ES
*Celtis cerasifera* C. K. Schneider	Ulmaceae	TB	P, S	MS
*Cerasus cerasoides* S. Y. Sokolov	Rosaceae	OR	P, S	ES
*Cerasus serrulata* Loudon	Rosaceae	FR, OR	P, S	ES
*Choerospondias axillaris* B. L. Burtt & A. W. Hill	Anacardiaceae	FR, TB	P, S	MS
*Crataegus scabrifolia* Rehder	Rosaceae	FR, MD, TB	P, S	MS
*Cyclobalanopsis lamellosa* Oersted	Fagaceae	CC, TB	P	LS
*Diospyros kaki* var. *silvestris* Makino	Ebenaceae	FR, TB	P, S	MS
*Elaeocarpus lanceifolius* Roxburgh	Elaeocarpaceae	MD, TB	P	LS
*Heynea trijuga *Roxburgh	Meliaceae	MD	P	MS
*Ilex polyneura* S. Y. Hu	Aquifoliaceae	TB	P	LS
*Ligustrum lucidum* W. T. Aiton	Oleaceae	FD, MD	P, S	MS
*Lindera communis* Hemsley	Lauraceae	FW, OL, OR, TB	P, S, A	MS
*Lindera megaphylla* Hemsley	Lauraceae	OL, TB	P	LS
*Lindera thomsonii* C. K. Allen	Lauraceae	OL, TB	P, S	MS
*Lithocarpus hancei* Rehder	Fagaceae	CC, TB	P	LS
*Litsea mollis* Hemsley	Lauraceae	MD, OL	P, S	MS
*Machilus rufipes* H. W. Li	Lauraceae	TB	P	LS
*Machilus yunnanensis* Lecomte	Lauraceae	OL, TB	P, S	LS
*Manglietia hookeri* Cubitt & W. W. Smith	Magnoliaceae	OR, TB	P	LS
*Michelia doltsopa* Buchanan-Hamilton ex Candolle	Magnoliaceae	OR, TB	P	LS
*Myrsine semiserrata* Wallich	Myrsinaceae	FW	P, S	MS
*Padus wilsonii* C. K. Schneider	Rosaceae	TB	P	MS
*Polyspora longicarpa* C. X. Ye ex B. M. Barthol. & T. L. Ming	Theaceae	OR	P	LS
*Quercus acutissima* Carruthers	Fagaceae	FD, FW, TB	S, A	MS
*Schima wallichii* Korthals	Theaceae	TB	P, S	MS
*Sorbus corymbifera* N. T. Kh’ep & G. P. Yakovlev	Rosaceae	FW	P, S	MS
*Symplocos paniculata* Miquel	Symplocaceae	OL, OR	P, S	MS
*Tetradium ruticarpum* T. G. Hartley	Rutaceae	MD	P	MS
*Turpinia cochinchinensis* Merrill	Staphyleaceae	FW	P	MS

^a^CC, charcoal; FD, fodder; FR, fruit; FW, firewood; MD, medicine; OL, oil; OR, ornamental; TB, timber.

^b^P, primary forest; S, secondary forest; A, agroforestry system.

^c^ES, early-successional; MS, mid-successional; LS, late-successional.

### Nursery experiment

An experimental nursery adjacent to the reserve was built to assess responses to propagation and to supply seedlings. Seeds from natural forest were sown in germination trays using a medium of loess and humus mixture and watered daily. Conditions in the nursery were similar to standard nursery practices in this region (i.e. ambient temperature 15 to 25°C, approximately 50% full sunlight). The number of germinated seeds was recorded each day until no additional seeds germinated. Our previous research [50] indicated that species such as *Lindera communis*, *Lindera thomsonii*, *Machilus rufipes*, *Myrsine semiserrata*, and *Ilex polyneura* have dormant seeds. However, only limited information is available on seed dormancy mechanisms and pre-germination treatment techniques for the studied species. Subsequently, potted seedlings with fully developed root systems were cultivated. The average planting size of seedlings was 25 cm in height. For further details of the nursery conditions, see Lu et al. [50].

### Field experiment

We evaluated the field performance of the selected species based on data from a restoration planting trial. The planting site, which was in the buffer zone of the reserve 500 m from the natural forest, was at an elevation of 2,140 m with alfisol as the dominant soil and a slope of 16 to 25°. Originally, the degraded land had been covered with subtropical evergreen broad-leaved forest, and was cleared by farmers between 1970 and 1983 to grow maize. The area was officially abandoned after the reserve was created in 1983, although sporadic grazing occurred until 1995. At the time of planting, the vegetation was dominated by dense grasses such as *Pteridium revolutum*, *Imperata cylindrica*, *Art*emisia *indica*, and *Ageratina adenophora* (an invasive species). Some pioneer shrub species (e.g. *Hypericum henryi*, *Viburnum cylindricum*, *Rubus* spp.) occurred in patches. The site was prepared by clearing shrubs and herbs within the planting line and digging planting holes (40 × 40 × 40 cm) at 2 × 2 m spacing before tree planting. Nursery-raised seedlings were outplanted in a randomly assigned order; location of each species was chosen by random draw, and species from different successional stages were mixed. Due to the availability of seedlings, approximately 45 individuals per species were planted in the same experimental site. Altogether 1,555 seedlings of 34 species at a density of nearly 2,500 seedlings per hectare were planted in August 2006, which is during the wet season. Each seedling was watered at the time of planting and three times during the dry season at 2-month intervals (December, February, April). After planting, hand weeding was carried out three times (May, July, September) a year. Immediately after the May weeding and therefore before the rainy season, seedlings were fertilized with 100 g of compound fertilizer around the stem. Seedling survival and seedling height were recorded every three months over a two-year period.

### Data analysis

The germination rate was calculated for each species. The survival rate was calculated for each species as the percentage of initially planted seedlings still alive 2 years after planting [27, 35]. Seedling growth was calculated as mean monthly height increments (ΔH, cm/month), as follows [16, 34]:

$$\Delta H=\frac{{height}_{final}-{height}_{initial}}{24}$$

Species with fewer than five individuals at the end of the experiment were excluded from the growth analysis. The growth rates of seedlings with negative growth were recorded as zero. Species performance standards were adapted from Elliott et al. [13] and Lu et al. [50] based on local conditions (Table 2). Each species was given a composite rating based on their combined scores for germination rate, survival rate, and mean monthly height growth. The species were divided into four categories: excellent, good, marginal, and poor. Analysis of variance (ANOVA) was used to detect differences in performance of species by the successional status. Next, multiple comparisons were conducted using Tukey’s HSD (Honestly Significant Difference) method (*p* = 0.05). All statistical analyses were conducted in R (R version 3.1.2) [60].

**Table 2:** Species performance standards.

Aspect	Categories	Score
**Germination rate**	>80%	3
	50–80%	2
	<50%	1
**Survival rate**	>70%	3
	50–70%	2
	<50%	1
**Mean monthly height growth**	>3 cm/month	3
	1–3 cm/month	2
	<1 cm/month or null	1
**Species rating**	Excellent	8–9
	Good	6–7
	Marginal	5
	Poor	3–4

## Results

### Germination

Germination rates ranged from 15 to 96% across species (Table 3). Of 34 species examined, 25 species had a germination rate greater than 50% and 12 species had a germination rate greater than 80%. Only one species (*Crataegus scabrifolia*) had a germination rate of less than 30%.

**Table 3:** Seed germination rates, seedling survival rates and growth performance of 34 native tree species from a subtropical evergreen broad-leaved forest in SW China.

Species	Germination (%)	Survival (%)	ΔH (cm/month) Mean (SE)	Rating
*Acer oliverianum*	42	60	1.78 (0.46)	Marginal
*Actinodaphne obovata*	67	0	-	Poor
*Alcimandra cathcartii*	70	4	-	Poor
*Alnus nepalensis*	48	94	8.35 (0.45)	Good
*Betula alnoides*	54	58	5.85 (0.50)	Good
*Celtis cerasifera*	33	44	0.51 (0.27)	Poor
*Cerasus cerasoides*	85	87	4.13 (0.34)	Excellent
*Cerasus serrulata*	95	92	6.64 (0.45)	Excellent
*Choerospondias axillaris*	68	55	2.38 (0.31)	Good
*Crataegus scabrifolia*	15	76	2.88 (0.33)	Good
*Cyclobalanopsis lamellosa*	79	20	1.79 (0.61)	Marginal
*Diospyros kaki* var. *silvestris*	73	97	2.92 (0.26)	Good
*Elaeocarpus lanceifolius*	45	16	-	Poor
*Heynea trijuga*	87	90	0.99 (0.11)	Good
*Ilex polyneura*	45	78	3.91 (0.52)	Good
*Ligustrum lucidum*	61	93	0.94 (0.16)	Good
*Lindera communis*	52	92	2.34 (0.13)	Good
*Lindera megaphylla*	90	8	-	Marginal
*Lindera thomsonii*	78	90	1.38 (0.16)	Good
*Lithocarpus hancei*	56	28	1.64 (0.51)	Marginal
*Litsea mollis*	40	12	-	Poor
*Machilus rufipes*	96	72	1.68 (0.16)	Excellent
*Machilus yunnanensis*	85	84	1.53 (0.15)	Excellent
*Manglietia hookeri*	70	39	1.30 (0.29)	Marginal
*Michelia doltsopa*	90	91	4.46 (0.25)	Excellent
*Myrsine semiserrata*	91	36	0.82 (0.20)	Marginal
*Padus wilsonii*	81	71	0.85 (0.13)	Good
*Polyspora longicarpa*	85	16	-	Marginal
*Quercus acutissima*	88	90	1.62 (0.17)	Excellent
*Schima wallichii*	69	36	2.29 (0.41)	Marginal
*Sorbus corymbifera*	96	94	2.77 (0.25)	Excellent
*Symplocos paniculata*	40	48	1.39 (0.30)	Poor
*Tetradium ruticarpum*	75	53	0.80 (0.18)	Marginal
*Turpinia cochinchinensis*	44	0	-	Poor

Seedling survival rates and mean monthly height increments (ΔH) were calculated at 2 years after planting. If a species had fewer than five individuals at that time, the columns corresponding to ΔH were left blank. Each species was given a composite rating (Table 2) based on their combined scores for germination rate, survival rate, and mean monthly height growth.

### Survival

Seedling survival rates after 2 years ranged from 0 to 97% across the studied species (Table 3). Survival rates were greater than 50% for 20 species and 16 species had survival rates greater than 70%. Pioneer species such as *Alnus nepalensis*, *Cerasus cerasoides*, and *Cerasus serrulata* had survival rates of greater than 85%. However, the survival rate of the pioneer *Betula alnoides* was only 58% and the mid to late-successional species *Actinodaphne obovata* and *Turpinia cochinchinensis* had zero survival at the end of 2 years. Overall, mid-successional species established well, with an acceptable mean survival rate of 63% (Table 4). Although the mean survival rate of late successional species (38%) was significantly lower (*p*<0.05) than pioneer species (83%), the seedlings of some late-successional species, including *Ilex polyneura*, *Machilus rufipes*, *Machilus yunnanensis*, and *Michelia doltsopa*, had greater than 70% survival rates.

**Table 4:** Nursery and field performance of species from different successional stages.

Successional status	Species number	Germination (%)		Survival (%)		MHI (cm/month)	
		Mean	SE	Mean	SE	Mean	SE
**Early-**	4	70.50^a^	11.51	82.75^a^	8.38	6.16^a^	0.24
**Mid-**	18	62.94^a^	5.46	63.17^ab^	7.02	1.74^b^	0.07
**Late-**	12	73.17^a^	5.03	38.00^b^	9.76	2.48^c^	0.14

Values with the different superscripts are significantly different (ANOVA, Tukey post hoc analysis, *p*<0.05); MHI: monthly height increments.

### Growth

As seven species had less than five surviving individuals at the end of the experiment, we only computed growth rates for 27 out of the 34 species. Mean monthly growth increments varied substantially between species (Table 3). Six species had height growth exceeding 3 cm/month, 15 species had growth rates from 1 to 3 cm/month, while six had growth rates below 1 cm/month. The four early-successional species grew rapidly. *Alnus nepalensis* grew fastest, attaining mean monthly height increments of more than 8 cm over the first 2 years. Two late-successional species also had high monthly height growth (*Michelia doltsopa*, 4.46 ± 0.25 cm/month and *Ilex polyneura*, 3.91 ± 0.52 cm/month). There were significant differences among species of different successional status in monthly height increments (ANOVA, *p*<0.001, Table 4).

### Species rating

Seven species were ranked as excellent species and eleven as good species in this study (Table 3). The four early-successional species performed well with relatively high survival and fast growth. Three late-successional species (*Machilus rufipes*, *M*. *yunnanensis*, and *Michelia doltsopa*) and two mid-successional species (*Sorbus corymbifera* and *Quercus acutissima*) also showed excellent performance. Of the 11 species ranked as good, eight species were mid-successional. Nine species showed marginal performance, and the seven remaining species performed poorly.

## Discussion

### Species nursery performance

When selecting species for forest restoration, it is important to choose species which can be successfully propagated using technology that is appropriate for the local context [28, 61]. Most of the 34 native species we examined had an acceptable seed germination rate using simple and low-cost techniques that are common in nurseries in China. Whereas tropical tree species often have recalcitrant seeds that are produced intermittently, making propagation more challenging and costly [62], our results suggest it may be relatively easy to propagate many native species from subtropical evergreen broad-leaved forests. Currently, most seedlings produced in China’s restoration nurseries are fast-growing timber species grown with sophisticated propagation techniques, including exotic species [50, 63]. To improve the quality of restoration plantings in China, production of more seedlings from a wider variety of native species is necessary.

### Species field performance

Survival and growth rates are important factors in selecting tree species for restoration activities [27, 64]. We examined the early survival and growth of native species seedlings in a degraded site which was dominated by under-shrubs and grasses. The light-demanding early successional species (including *Alnus nepalensis*, *Betula alnoides*, *Cerasus cerasoides*, and *Cerasus serrulata*) performed well with high survival rates and rapid growth. These species can reduce the costs of restoration efforts and provide forest products and services in a short period of time. However, diversity is likely to remain low unless these species are mixed with later successional species.

We found that mid-successional and late-successional species had slower growth and lower survival rates, but with a large degree of variation among species. Our study shows that some mid-successional species, such as *Heynea trijuga*, *Lindera communis*, *Quercus acutissima*, and *Sorbus corymbifera* are suitable for restoration of highly degraded sites. Meanwhile, some late-successional species, such as *Ilex polyneura*, *Machilus rufipes*, *Machilus yunnanensis*, and *Michelia doltsopa* are also sound choices. Similarly, several previous studies conducted in tropical areas have demonstrated that some later successional species can perform well in open sites (e.g. [27, 34, 65, 66]). These findings indicate that with careful selection, some later successional species can be incorporated into restoration plantings. However, the other later successional species in our study performed poorly with low survival rates and slow growth, including several species (*Alcimandra cathcartii*, *Cyclobalanopsis lamellosa*, and *Lithocarpus hancei*) that are dominant in the nearby natural forest [41, 59]. Further research is needed in order to determine whether the survival and growth rates of these species can be enhanced using methods such as larger seedlings, direct seeding, inoculation with mycorrhizal fungi, increased post-planting care, planting in less degraded sites or planting after the establishment of a pioneer canopy [31, 34, 67]. As trees are long-lived organisms, the effectiveness and cost-benefit of restoration treatments and the implications for later forest management need long-term field research.

### The role of species from different successional stages

As ecological information on most native subtropical tree species is still sparse, this study shows that species successional guilds can be used as simple guidelines for species selection. Fast-growing native timber species such as *Alnus nepalensis* (a nitrogen-fixing tree) can act as nurse trees for other species in highly degraded sites, and also enhance the business case for restoration by providing early revenues through selective thinning and sustainable harvesting [16, 39, 68]. Currently in China, exotic species (e.g. *Acacia* spp.) are often promoted as nurse tree species in forest restoration practices [69]. However, our results suggest that native pioneer tree species may be a more appropriate choice for restoration efforts, especially those that are also useful timber species, such as *Alnus nepalensis*.

Our results also indicate that certain mid- and late-successional species (such as *Lindera communis*, *Quercus acutissima*, *Machilus rufipes*, *Machilus yunnanensis*, and *Michelia doltsopa*) have high potential for restoration planting in this region. Mid- and late-successional species are essential for maintaining productivity, species richness and ecological services in forest ecosystems [25, 70, 71]. As forest restoration is a long-term process, long-lived, slower-growing tree species are better suited to long-term carbon sequestration than fast-growing, short-lived species [71, 72]. In degraded landscapes, forest fragmentation and defaunation limit the dispersal of seeds, particularly larger seeds, and as a result, natural regeneration of later successional species is often limited [73–76]. Passive secondary succession is likely to be very slow in many degraded sites and the resulting forest is often dominated by pioneer species, which results in a landscape with little ecological and conservation value [68, 77]. Hence, active planting of later successional species is often required to accelerate forest succession and avoid the creation of “pioneer deserts” [30].

Furthermore, later successional species often have higher market values than fast-growing pioneer species, and as supply shortages become more common, their market values are increasing [9]. At our study site, local people continue to harvest the wood of valuable later successional species such as *Machilus yunnanensis* and *Michelia doltsopa* from nearby natural forests, contributing to further degradation. Some studies have shown that forest restoration around remaining natural forests (e.g. isolated protected areas) can balance the benefits of biodiversity conservation and rural livelihoods [78]. Incorporating economically attractive native species into local restoration plantings may incentivize local people to protect and maintain them. At the same time, producing alternative sources of timber may reduce over-harvesting and disturbance in natural forest.

### Candidate species for restoration

Based on our study, we propose 18 species with excellent and good performance as candidates for restoration plantings in this area. Of these species, *Alnus nepalensis*, *Betula alnoides*, *Choerospondias axillaris*, *Ligustrum lucidum*, *Lindera communis*, *Machilus yunnanensis*, and *Quercus acutissima* have been used in previous reforestation projects in this region, but only on a small scale [79–81]. Among them, *Alnus nepalensis*, *Lindera communis*, and *Quercus acutissima* are also employed in traditional agroforestry systems in the region. *Alnus nepalensis* has been planted into swidden fallows for centuries, both because it is a valuable source of timber and because it enhances soil quality through nitrogen fixation [80]. *Lindera communis* is a good timber and roadside tree, and its fruit can also produce high-quality oil and attract small birds in the study area [81]. Firewood harvesting is regarded as a major and chronic driver of forest degradation in densely populated areas [82], and *Quercus acutissima* can provide a sustainable source of firewood for locals, as well as reducing soil erosion [79]. Restoration with these multi-purpose species could provide economic and ecological benefits to local people, while restoration success could be enhanced by traditional management experience. Ambitious forest restoration targets can best be achieved through addressing local needs and involving local communities in species selection [71].

Our results offer empirical evidence that some previously ignored native subtropical species have the potential to play important roles in restoration planting in China. Further studies could evaluate more species and biomes, while information on tree species performance could be made available through user-friendly platforms such as the World Agroforestry Centre’s online database “Agroforestry Species Switchboard” and the mobile application “vegetationmap4africa” [83]. More importantly, participatory approaches should be applied to promote the use of indigenous knowledge and involve the local community in species selection [84, 85]. This could enhance information exchange between researchers and practitioners, and enable both local farmers and policymakers to identify and use appropriate native tree species in reforestation and restoration projects [1, 6]. Further research integrating both scientific and indigenous knowledge is also required to investigate the implications of interactions between native species for multiple species planting [32, 85, 86].

The indicators used in this study (i.e. germination, survival, and growth) are relatively measurable and actionable; furthermore, the use of species successional status to aid species selection for forest restoration is a flexible and generalizable tool. The adoption of the approach described in this study, combined with the full participation of local communities, could enable those involved in restoration to select species more reliably and to design their restoration plans using a range of suitable species, including previously neglected native species. This could improve the success of restoration activities in terms of cost, local income generation, enhanced biodiversity and provision of ecosystem services in the subtropics and elsewhere.
